# Amphioxus *SYCP1*: a case of retrogene replacement and co-option of regulatory elements adjacent to the ParaHox cluster

**DOI:** 10.1007/s00427-017-0600-9

**Published:** 2018-01-02

**Authors:** Myles G. Garstang, David E. K. Ferrier

**Affiliations:** 1 The Scottish Oceans Institute, Gatty Marine Laboratory, University of St Andrews, East Sands, St Andrews, Fife, KY16 8LB UK; 2 Present Address: School of Biological Sciences, University of Essex, Wivenhoe, Colchester, Essex, CO4 3SQ UK

**Keywords:** Amphioxus, ParaHox, Retrogene replacement, SYCP1, Synaptonemal complex, *CHIC* genes

## Abstract

Retrogenes are formed when an mRNA is reverse-transcribed and reinserted into the genome in a location unrelated to the original locus. If this retrocopy inserts into a transcriptionally favourable locus and is able to carry out its original function, it can, in rare cases, lead to retrogene replacement. This involves the original, often multi-exonic, parental copy being lost whilst the newer single-exon retrogene copy ‘replaces’ the role of the ancestral parent gene. One example of this is amphioxus *SYCP1*, a gene that encodes a protein used in synaptonemal complex formation during meiosis and which offers the opportunity to examine how a retrogene evolves after the retrogene replacement event. *SYCP1* genes exist as large multi-exonic genes in most animals. *AmphiSYCP1*, however, contains a single coding exon of ~ 3200 bp and has inserted next to the ParaHox cluster of amphioxus, whilst the multi-exonic ancestral parental copy has been lost. Here, we show that *AmphiSYCP1* has not only replaced its parental copy, but also has evolved additional regulatory function by co-opting a bidirectional promoter from the nearby *AmphiCHIC* gene. *AmphiSYCP1* has also evolved a de novo, multi-exonic 5′untranslated region that displays distinct regulatory states, in the form of two different isoforms, and has evolved novel expression patterns during amphioxus embryogenesis in addition to its ancestral role in meiosis. The absence of ParaHox-like expression of *AmphiSYCP1*, despite its proximity to the ParaHox cluster, also suggests that this gene is not influenced by any potential pan-cluster regulatory mechanisms, which are seemingly restricted to only the ParaHox genes themselves.

**Electronic supplementary material:**

The online version of this article (10.1007/s00427-017-0600-9) contains supplementary material, which is available to authorized users.

## Introduction

Synaptonemal complex protein 1 (SYCP1) belongs to a group of proteins that form the synaptonemal complex, which is crucial to the process of meiotic recombination (Page and Hawley 2004; Zickler and Kleckner 1999). Specifically, SYCP1 forms the transverse filaments that link the lateral elements of the complex and is made up of coiled-coil domains (Meuwissen et al. 1992). Such is the propensity of this protein to form these ordered transverse filaments that SYCP1 has been observed to form synaptonemal-like structures ex vivo, forming ‘polycomplexes’ made up of stacks of transverse filaments (Liu et al. 1996), highlighting this important structural role. Indeed, in *SYCP1−/−* mice, meiotic synapses are unable to form and meiosis does not progress. Whilst previously only identified within the vertebrates, work carried out by Fraune et al. (Fraune et al. 2012a) has shown *SYCP1* to be more ancient and widely conserved across the Metazoa, with *SYCP1* present in the cnidarian *Hydra vulgaris*, the poriferan *Amphimedon queenslandica* and the ctenophore *Pleurobrachia pileus*.

Due to its crucial role in meiosis, *SYCP1* expression is observed within the germ cells of vertebrates (Iwai et al. 2006; Zheng et al. 2009) and also within the basal-most cells of the *Hydra* testis, highlighting this deeply conserved role of SYCP1 in meiosis (Fraune et al. 2012a). This localisation of SYCP1 to the germ cells is such that a promoter fragment of *SYCP1* is sufficient to drive germline expression in zebrafish without requiring additional regulatory elements (Gautier et al. 2013). It is this expression of *SYCP1* within the germ cells that may have led to the multiple instances of *SYCP1* retrogene formation (Ferrier et al. 2005; Sage et al. 1997), with the mouse alone having at least one *SYCP1* retrocopy. The first of these, *Sycp1-ps1*, is present across several related *Mus* sub-species but has accumulated multiple point mutations and deletions and is no longer transcribed. The second, *Sycp1-ps2,* is transcribed and represents a much younger pseudogene. Interestingly, this second retrocopy is found only within lab strains of *Mus musculus* and is absent even from wild *Mus musculus* populations, highlighting the much more recent nature of this second *Sycp1* retrotransposition event (Sage et al. 1997).

The role of SYCP1 in meiosis makes it a candidate for the ‘out-of-testis’ route of retrogene production, in which functional retrogenes often emerge from genes expressed within the testis, whether there is function within the testis or not (Kleene et al. 1998). Indeed, most genes that give rise to retrogenes are often found to be originally expressed within the testis (Marques et al. 2005; Vinckenbosch et al. 2006), and it seems that many retrogenes may even be initially transcribed within the testis before gaining additional functions, due to the promiscuous transcription in this tissue. Many examples exist of retrogenes becoming bona fide functional genes that play important roles. The FGF4 retrogene present in short-legged dog breeds is responsible for the common occurrence of chondrodysplasia in these breeds (Parker et al. 2009), whilst the vertebrate RHOB gene plays a role as a tumour suppressor gene (Prendergast 2001) and originates from a retrotransposition early in vertebrate evolution (Sakai et al. 2007). Indeed, severe disease phenotypes can arise from mutations in retrogenes, such as the gelatinous drop-like corneal dystrophy arising from mutations that disrupt *TACTSTD2*, which leads to blindness (Tsujikawa et al. 1999), or the deletion of the retrogene UTP14B which leads to a severe recessive defect in spermatogenesis (Bradley et al. 2004). In some cases, the retrogene has not only become functional or retained functionality, but has also replaced the parental gene copy in function and resulted in the loss of the parental copy from the genome. This phenomenon, known as retrogene replacement (Krasnov et al. 2005), or alternatively as ‘orphaned retrogenes’ (Ciomborowska et al. 2013), has been documented largely by single-exon gene copies lying in a different locus from that of the ancestral multi-exonic parent copy. Though few in number, studies of retrogene replacement can give unique insight into the regulatory environment of genes and genomic loci. This is especially true in the case of the *Iroquois-Sowah* locus of Bilateria (Maeso et al. 2012), in which the ancestrally linked *Iroquois* and *Sowah* genes have been decoupled in the tetrapods. Despite this syntenic block being maintained for over 600 million years of evolution, the parental *Sowah* gene has been pseudogenised and lost from the *Iroquois* locus in tetrapods, whilst a retrogene copy now exists elsewhere in the genome. Interestingly, *Iroquois* cis-regulatory modules remain within the pseudogenised remnants of *Sowah* genes next to the *Iroquois* locus (Maeso et al. 2012).

*SYCP1* is a gene that has undergone retrogene formation in multiple chordate lineages but has also undergone retrogene replacement within the cephalochordate amphioxus (Ferrier et al. 2005), making this a particularly intriguing case. Here, a single-exon retrogene copy of *SYCP1* has inserted just upstream of the ParaHox gene *Gsx*, a feature unique to the amphioxus ParaHox cluster. Though previously named *AmphiSCP1* by Ferrier et al. (2005), this amphioxus *SYCP1* gene will be amended to *AmphiSYCP1* here, in order to maintain consistency across species in light of the detailed analysis by Fraune et al. (2012a). With no multi-exonic copy present elsewhere in the amphioxus genome, it appears that the single-exon *AmphiSYCP1* retrogene is the only *SYCP1* gene present in amphioxus (Ferrier et al. 2005). The ParaHox cluster of chordates has previously been shown to be open to invasion by retrotransposons (Osborne and Ferrier 2010; Osborne et al. 2006), perhaps due to *Cdx* transcription within the germline opening up the cluster to transposable elements (Kurimoto et al. 2008). With complex and perhaps long-range or even pan-cluster regulatory mechanisms directing ParaHox gene expression across the cluster (Garstang and Ferrier 2013; Osborne et al. 2009), *AmphiSYCP1* presents an excellent case with which to study how the regulation and expression of retrogenes are affected when they enter a new locus. The likely dense regulatory landscape of the amphioxus ParaHox cluster provides an opportunity to examine both the regulation of *AmphiSYCP1* and the surrounding genes.

Here, we show that *SYCP1* underwent retrogene replacement prior to the divergence of the *Branchiostoma* lineage of amphioxus, with the *AmphiSYCP1* retrogene inserting adjacently to the amphioxus ParaHox cluster. Despite its proximity to the ParaHox gene *Gsx*, *AmphiSYCP1* does not display ParaHox-like expression but does display embryonic expression in addition to the expected gonadal expression, which is altogether atypical for a gene family whose only known role is in meiosis. Identification of a transcribed multi-exonic *AmphiSYCP1* 5′ untranslated region (UTR) with multiple isoforms suggests the de novo evolution of a regulatory 5′ UTR after retrogene insertion. Finally, the proximity of the *AmphiSYCP1* 5′ UTR to the 5′ of the adjacent *AmphiCHIC* gene, similar expression of *AmphiCHIC* to *AmphiSYCP1* and the high support for a bidirectional promoter region overlapping the transcriptional start site of these two genes suggest the co-option of regulatory information from *AmphiCHIC* by *AmphiSYCP1*.

## Results

### *AmphiSYCP1* is a retrogene adjacent to the ParaHox cluster that has led to retrogene replacement of the ancestral parental copy prior to the divergence of the *Branchiostoma*

**Fig. 1:**
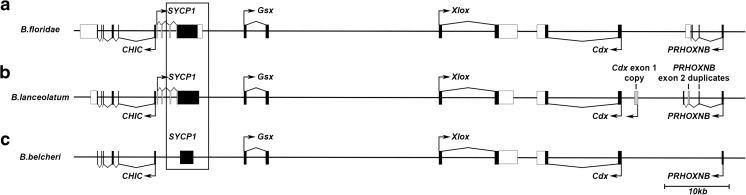
Comparison of *SYCP1* position across amphioxus species. **a** A schematic of the *B. floridae* SYCP1 gene with relative positions of coding sequence and identified 5′ and 3′ UTRs with respect to the surrounding genes. **b** A schematic of the *B. lanceolatum* SYCP1 gene with relative positions of coding sequence with respect to the surrounding genes. **c** A schematic of the *B. belcheri* SYCP1 gene with relative positions of coding sequence with respect to the surrounding genes. *B. belcheri* SYCP1 is missing both the 3′ and 5′ ends of the coding sequence. Coding exons are represented in black, whilst UTR is represented in white. Chevron lines linking exons show the intron-exon structure of genes based on mRNA transcripts. Grey denotes artefacts resulting from genome scaffold assembly errors. Right-angle arrows indicate known transcriptional start sites and orientation of transcription

The previously identified *SYCP1* coding sequence (Ferrier et al. 2005) was used to confirm that *Branchiostoma floridae SYCP1* was indeed upstream of *Gsx* and present as a single coding exon within the *B. floridae* genome (Fig. 1a). Furthermore, *SYCP1* is also present in the same location and as a single coding exon, in both the *Branchiostoma lanceolatum* and *Branchiostoma belcheri* genomes (see the “Materials and methods” section) (Fig. 1b, c), revealing that the *SYCP1* retrotransposition event must have occurred prior to the divergence of the *Branchiostoma* genus. Comprehensive searches against genomic and transcriptomic databases from all three *Branchiostoma* species reveal no other *SYCP1* gene copies or transcripts bar the *AmphiSYCP1* retrogene. The single-coding-exon organisation of amphioxus *SYCP1* genes is in stark contrast to the multi-exonic arrangement of *SYCP1* genes in most other species (Table S1), consistent with the amphioxus gene originating via retrogene replacement.

Whilst the whole coding sequence for *SYCP1* is present in both *B. floridae* and *B. lanceolatum*, *B. belcheri* Sc0000020 contains only the central region of *SYCP1* coding sequence as the 5′ adjacent sequence does not match *SYCP1* and seems to be an unrelated non-coding sequence, and the 3′ adjacent sequence is represented by a string of N’s. This is likely due to the low-quality sequence in this region or problems with the assembly within v15h11.r2 rather than *B. belcheri SYCP1* being incomplete. The position of amphioxus *SYCP1* genes is given relative to the flanking *CHIC* and *Gsx* genes in Fig. 1 for *B. floridae* (Fig. 1a), *B. lanceolatum* (Fig. 1b) and *B.belcheri* (Fig. 1c).

### *AmphiSYCP1* is expressed during embryogenesis in addition to the expected expression within the adult gonads

**Fig. 2:**
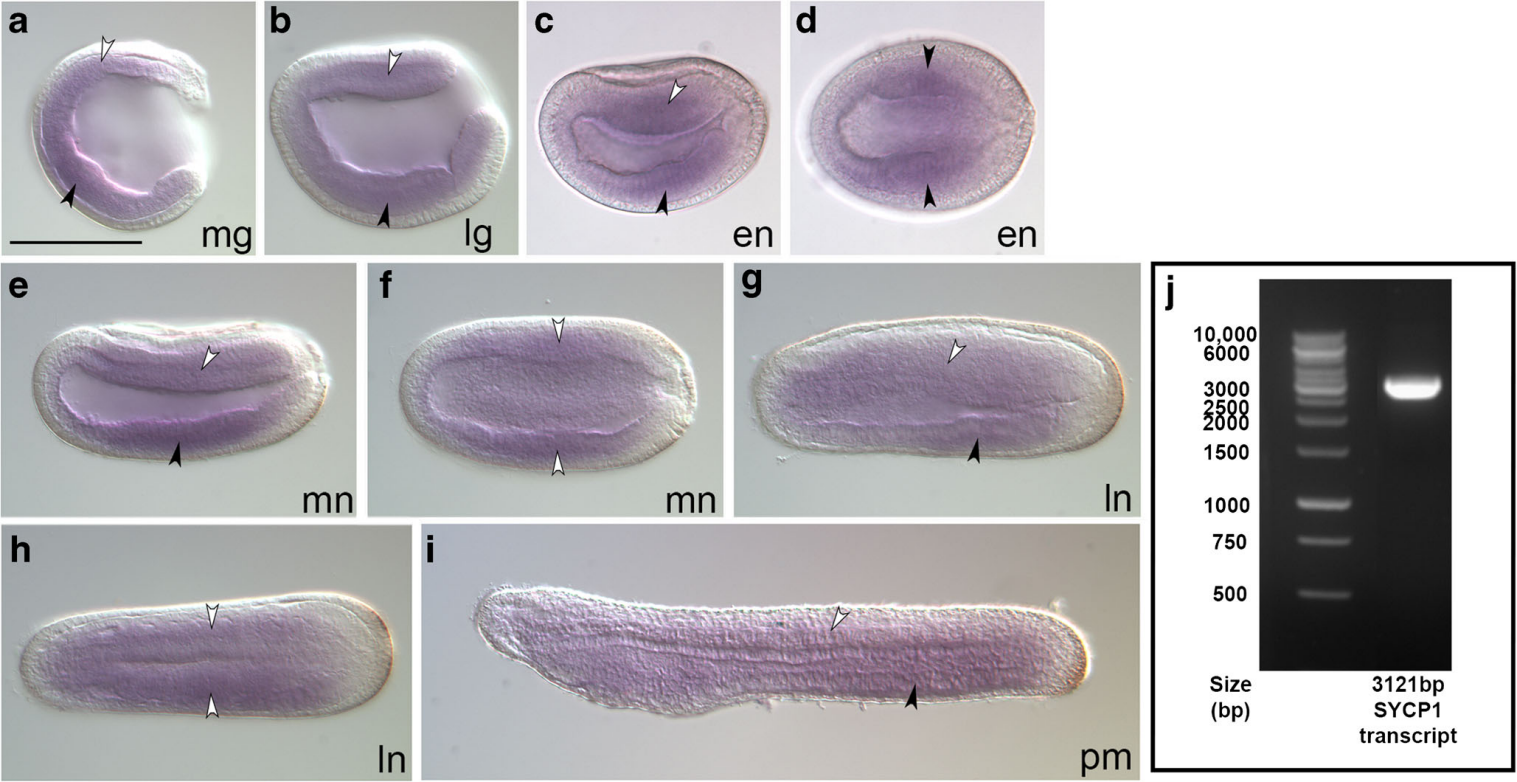
Expression of *B. lanceolatum SYCP1* transcripts within embryos and gonadal tissue. **a–i** Embryonic expression of *B. lanceolatum SYCP1* is shown from the mid-gastrula (**a**) to the pre-mouth (**i**) stages of development. Expression begins in the endoderm (black arrowheads) and dorsal mesoderm (white arrowheads) at the mid-gastrula stage to the late gastrula (**a**, **b**) before becoming more restricted to the centre of the animal and excluded from the extreme posterior in the early neurula (**c**, **d**). This expression pattern continues into the mid-late neurula (**e**, **f**). Expression reaches anteriorly to a region below the forming cerebral vesicle throughout the late neurula–pre-mouth (**g**–**i**), whilst expression elsewhere becomes much more diffuse throughout the somites and endoderm. **a**, **b**, **c**, **e**, **g**, **i** represent lateral views, whilst **d**, **f**, **h** represent dorsal views. **j** shows the 3121-bp *SYCP1* mRNA transcript cloned from *B. lanceolatum* gonadal total mRNA. mg mid-gastrula, lg late gastrula, en early neurula, mn mid-neurula, ln late neurula, pm pre-mouth. Scale bar represents 100 μm

In order to examine if *AmphiSYCP1* has come under the influence of any nearby ParaHox regulatory elements, in situ hybridisation of *AmphiSYCP1* was carried out on a time course of *B. lanceolatum* embryos, ranging from mid-gastrula to pre-mouth larvae, as these are the stages that display collinear ParaHox expression. This revealed extensive expression of *AmphiSYCP1* throughout the stages examined (Fig. 2a–i), within both the mesoderm and endoderm, though this expression does not show ParaHox-like collinearity (Osborne et al. 2009) and is much more extensive throughout the embryo than might be expected if *AmphiSYCP1* were being controlled by ParaHox regulatory elements. This expression is observed within the mid-gastrula (Fig. 2a) throughout the mesendoderm (black and white arrowheads) and continues into the late gastrula (Fig. 2b) where the mesendoderm is beginning to differentiate into the ventral endoderm (black arrowhead) and dorsal mesoderm (white arrowhead) but is absent from the ectoderm and neurectoderm in both stages. As embryogenesis progresses to the early neurula (Fig. 2c, d), expression is restricted more towards the central region of the embryo along the anterior-posterior axis, again present in the endoderm and mesoderm, but with expression notably absent from the extreme posterior of the embryo, ectoderm and developing neural plate. By the mid-late neurula stages (Fig. 2e, h), expression is undetectable in the posterior tailbud but still present throughout the central mesoderm (white arrowheads) and endoderm (black arrowhead). Expression remains absent from the ectoderm and neural tube. During the pre-mouth stage (Fig. 2i), *AmphiSYCP1* expression is present throughout the mesoderm and endoderm but absent from the posterior tailbud region, the ectoderm and the extreme anterior tip of the embryo. Finally, in addition to embryonic expression, *AmphiSYCP1* transcripts were cloned via RT-PCR from adult *B. lanceolatum* gonadal cDNA, confirming the expression of *AmphiSYCP1* within adult gonads as expected for a meiotic gene (Fig. 2j).

### *AmphiSYCP1* has evolved a de novo 5′ UTR with distinct isoforms

**Fig. 3:**
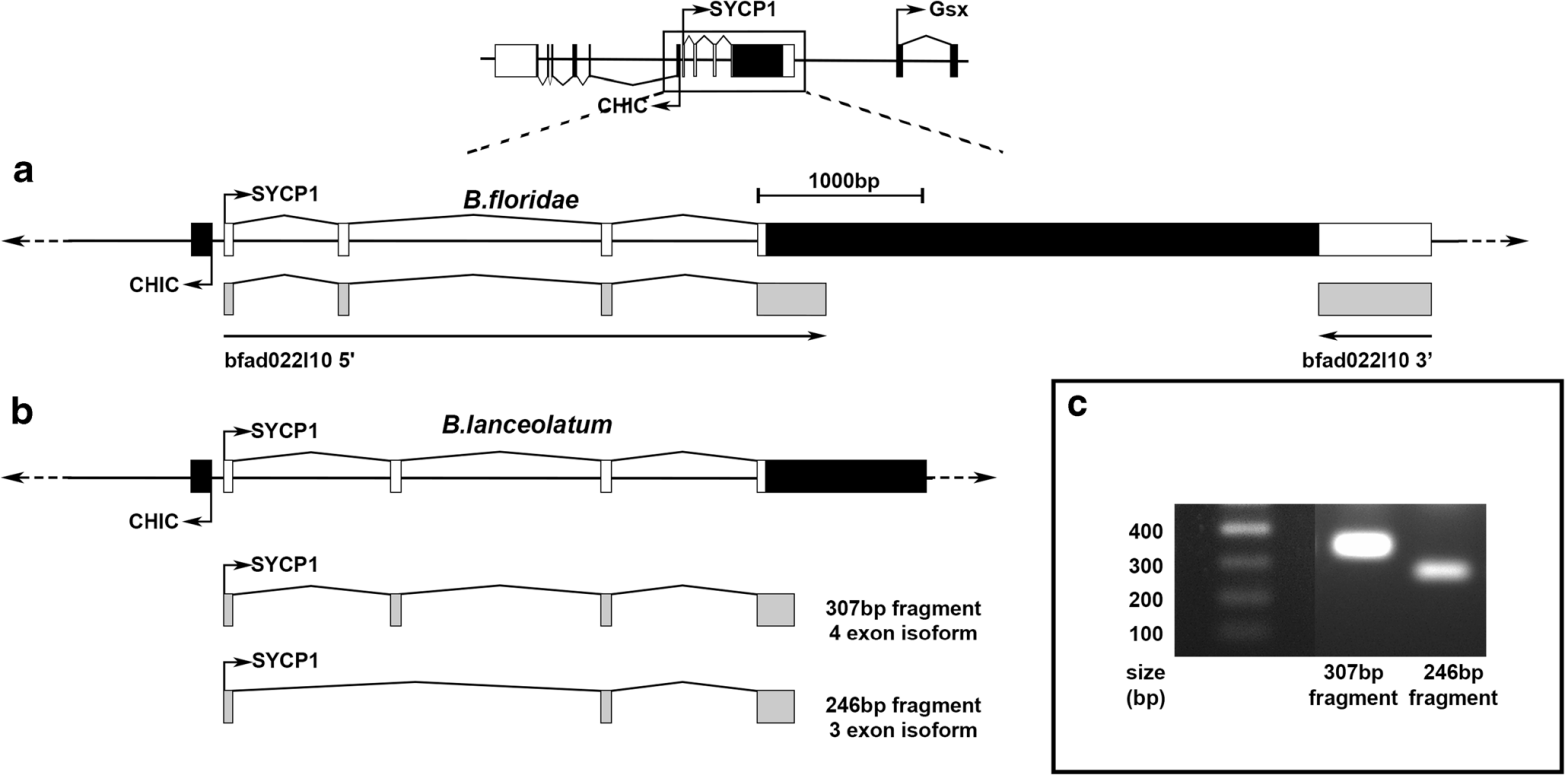
Amphioxus *SYCP1* has a multi-exonic 5′ UTR. A schematic depicting the relative positions of exons within the *CHIC-SYCP1* region of *B. floridae* and *B. lanceolatum* genomes. Black boxes represent coding sequence, white boxes represent UTR and grey boxes represent sequenced transcripts. Right-angle arrows indicate transcriptional start sites and orientation of transcription. **a** The *B. floridae* EST transcript bfad022|10 identifies both a multi-exonic 5′ UTR, as well as a 3′ UTR, that is adjacent to the single-exon *SYCP1* coding sequence. **b** Whilst *B. lanceolatum* also has a multi-exonic 5′ UTR, two different isoforms are present, one with four UTR exons whilst the second isoform lacks 5′ UTR exon 2. **c** Agarose gel depicting the two cloned *B. lanceolatum SYCP1* 5′ UTR fragments seen in schematic **b**. The longer is 307 bp in length, whilst the shorter is 246 bp in length. Reamplification of individual bands is shown to enable better visualisation of these independent clones. These fragments were obtained via PCR from *B. lanceolatum* gonadal cDNA

*B. floridae SYCP1* has previously been described as a retrogene, as it contains a single open reading frame with no introns within the amphioxus ParaHox PAC clones 33B4 and 36D2 (Ferrier et al., 2005). Searches for *B. floridae SYCP1* in the *B. floridae* expressed sequence tagged (EST) database (http://amphioxus.icob.sinica.edu.tw/) (Yu et al. 2008) revealed a *B. floridae* cDNA clone, bfad022l10, containing 5′ and 3′ ESTs that align to *B. floridae SYCP1* coding sequence and immediately flanking non-coding sequence (Fig. 3a). This EST clone was obtained from whole adult animal, which would be consistent with *SYCP1* expression within meiotic cells within the gonads.

The 3′ EST, bfad022l10 3′ (accession number BW716295.1), encompasses a 685-bp 3′ UTR downstream of the coding sequence of *SYCP1*. This represents a single exon containing the *SYCP1* coding sequence and 3′ UTR. As expected, the 5′ EST, bfad022l10 5′ (accession number BW697675.1), aligned to the most 5′ coding sequence of *B. floridae SYCP1*, with a 334-bp alignment covering this region. Additionally, a short 53-bp region immediately 5′ and adjacent to the coding sequence also matched the EST, designating 5′ UTR sequence present in the same exon as the coding sequence.

In addition, the 5′ EST, bfad022l10 5′, also aligned to further regions upstream of the *SYCP1* coding exon, with the mRNA sequence indicating three exons spread throughout the 3259 bp between the coding regions of *SYCP1* and *CHIC* (Fig. 3a). The three additional 5′ UTR exons were identified with discontiguous MegaBLAST, in order to accommodate sequence polymorphisms within these short exons relative to the genomic sequence. In total, only 16 nucleotides across the entire 599 bp of bfad022l10 5′ did not show a match to the *B. floridae* ParaHox genomic sequence.

In order to identify if this novel 5′ UTR was also present in *B. lanceolatum*, the EST data collected from *B. floridae* was then aligned to the *B. lanceolatum* ParaHox scaffold and primers designed against the beginning of 5′ UTR exon 1 and the 5′ of the coding region. These were used to clone the 5′ UTR region from adult *B. lanceolatum* gonadal cDNA. This not only isolated a transcribed, spliced *AmphiSYCP1* 5′ UTR transcript, but also identified two distinct isoforms of this transcript (Fig. 3b, c). The first of these is a long isoform (307-bp polymerase chain reaction (PCR) fragment) that contains all four *AmphiSYCP1* 5′ UTR exons, with exon 4 contiguous with the coding exon sequence. The second is a shorter isoform (246-bp PCR fragment) that lacks *AmphiSYCP1* 5′ UTR exon 2 but is otherwise identical to the longer isoform.

### *AmphiSYCP1* shares a bidirectional promoter with the adjacent *AmphiCHIC* gene

As the 5′ UTR of *AmphiSYCP1* must have evolved post-insertion of the ancestral amphioxus *SYCP1* single-exon retrogene, a promoter region driving the transcription of this 5′ UTR sequence must have either been co-opted from an existing nearby promoter sequence or evolved de novo. In order to establish which of these was the case, a total of 7000 bp, starting from within *AmphiCHIC* intron 1 to the end of the *AmphiSYCP1* coding exon, were analysed for promoter sequences. This ensured that the transcriptional start sites of *AmphiSYCP1* and its neighbour *AmphiCHIC* were both included as well as any possible overlap of promoter sequences. Three independent promoter prediction algorithms, Neural Network Promoter Prediction (NNPP), TSSW and ProScan1.7, were used across both *B. floridae* and *B. lanceolatum* to look for consistency across algorithms, which should increase confidence in the validity of any predicted promoters.

**Fig. 4:**
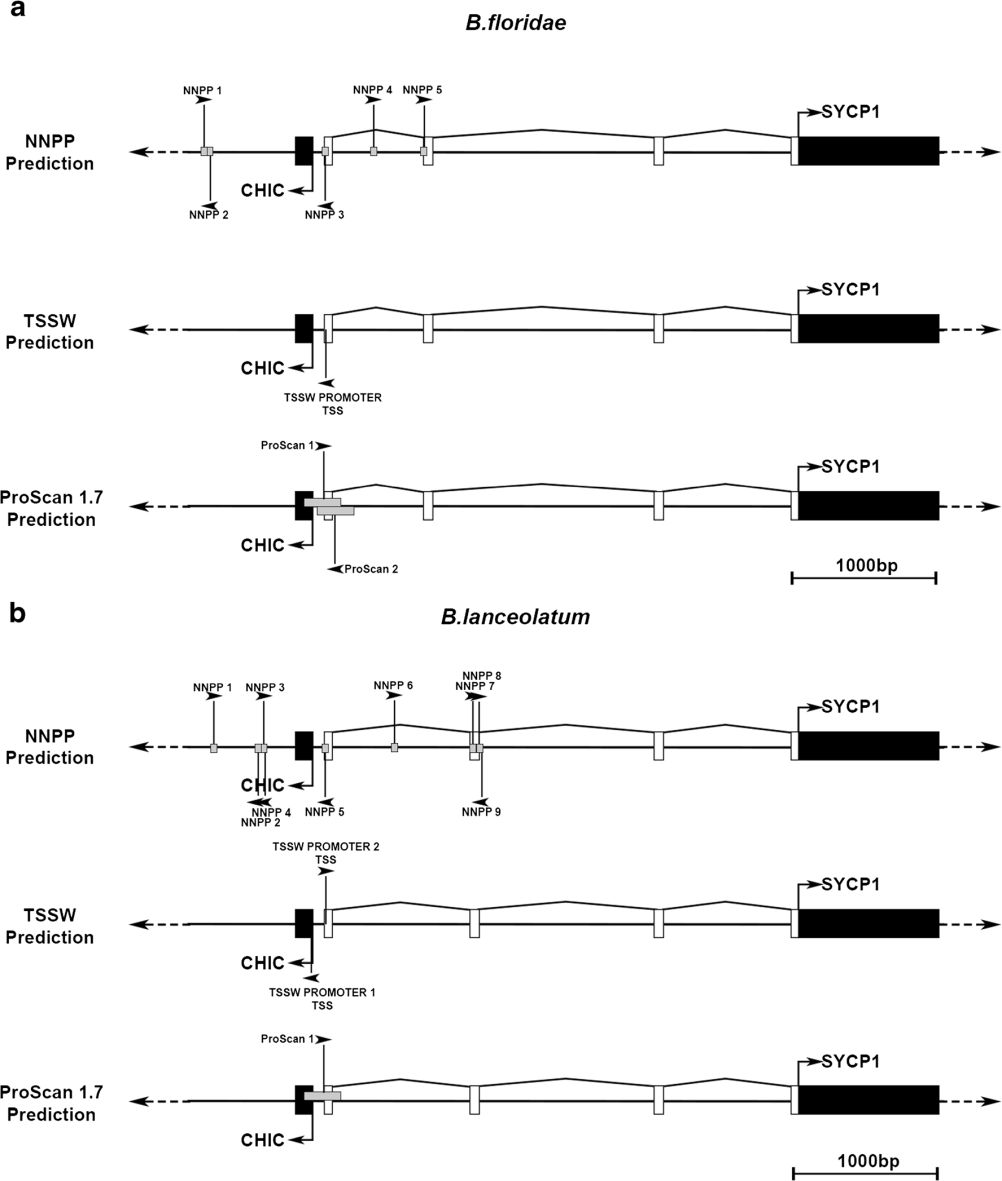
Promoter analysis of the *B. lanceolatum* and *B. floridae CHIC/SYCP1* loci. For the *SYCP1/CHIC* locus of both *B. lanceolatum* and *B. floridae*, promoter sequences predicted by either NNPP, TSSW or ProScan 1.7 are visualised relative to the surrounding *CHIC* and *SYCP1* exon-intron structures. The size and position of each predicted promoter identified are indicated by a grey box/black vertical line. In addition, black arrowheads indicate the direction of the DNA strand the promoter was identified upon. **a** For *B. floridae*, five promoters were predicted by NNPP (NNPP1–5), one by TSSW (TSSW promoter TSS) and two by ProScan 1.7 (ProScan1, ProScan 2). Only one promoter region, including NNPP3, TSSW promoter TSS, and ProScan 1 and 2, agrees across all three prediction models. **b** For *B. lanceolatum*, nine promoters were predicted by NNPP (NNPP1–9), two by TSSW (TSSW promoter 1 TSS, TSSW promoter 2 TSS), whilst only one was predicted by ProScan 1.7 (ProScan1). Only one promoter region, including NNPP5, TSSW promoter 1 TSS, TSSW promoter 2 TSS and ProScan 1, agrees across all three prediction models. This region also agrees across both *B. floridae* and *B. lanceolatum* (**a**, **b**), further supporting the confidence of this region as a bona fide promoter. In addition, this promoter is predicted in both directions within both species, suggesting a bidirectional promoter. Back boxes represent coding exons, whilst white boxes represent UTR exons. Right-angle arrows indicate known translational start sites and the orientation of transcription

Within *B. floridae*, a total of five 50-bp predicted promoter sequences were identified by NNPP (Fig. 4a) (Table S2), with the prediction with the highest support located surrounding the start of *AmphiSYCP1* 5′ UTR exon 1. This region, annotated as NNPP3 in Fig. 4a, was the only sequence predicted in all three Promoter prediction programs and had the highest support value in both NNPP and ProScan 1.7 (Table S2). This was also the only region predicted by TSSW and is identified as 50 bp in length using NNPP and 250 bp in ProScan. It also lies on the negative strand and spans the start of *AmphiSYCP1* 5′ UTR exon 1, in the same orientation as the *CHIC* gene, and is located 56 bp upstream of *AmphiCHIC* (Fig. 4a).

Within *B. lanceolatum*, a total of nine 50-bp predicted promoter sequences were identified by NNPP (Fig. 4b) (Table S2), with the prediction with the highest support again located surrounding the start of *AmphiSYCP1* 5′ UTR exon 1, annotated as NNPP5 in Fig. 4b. This region was also identified in TSSW (TSSW2) and overlaps a second TSSW hit facing in the opposite orientation towards *AmphiCHIC* (TSSW1). Finally, this region was identified in both NNPP and TSSW and is also identified as a 250-bp region oriented in the direction of *AmphiSYCP1* 5′ UTR in ProScan 1.7. This ProScan-predicted promoter also overlaps the first exons of both *AmphiCHIC* and *AmphiSYCP1* 5′ UTRs.

All three prediction programs thus agree on a strong candidate promoter region that overlaps the first exons of both *AmphiCHIC* and *AmphiSYCP1* 5′ UTRs. Also, transcription is predicted from this region in both directions in both species. This implies that *AmphiSYCP1* has co-opted a promoter with bidirectional capability from the neighbouring *AmphiCHIC* gene.

### *AmphiCHIC* is expressed in the same tissues as *AmphiSYCP1*

**Fig. 5:**
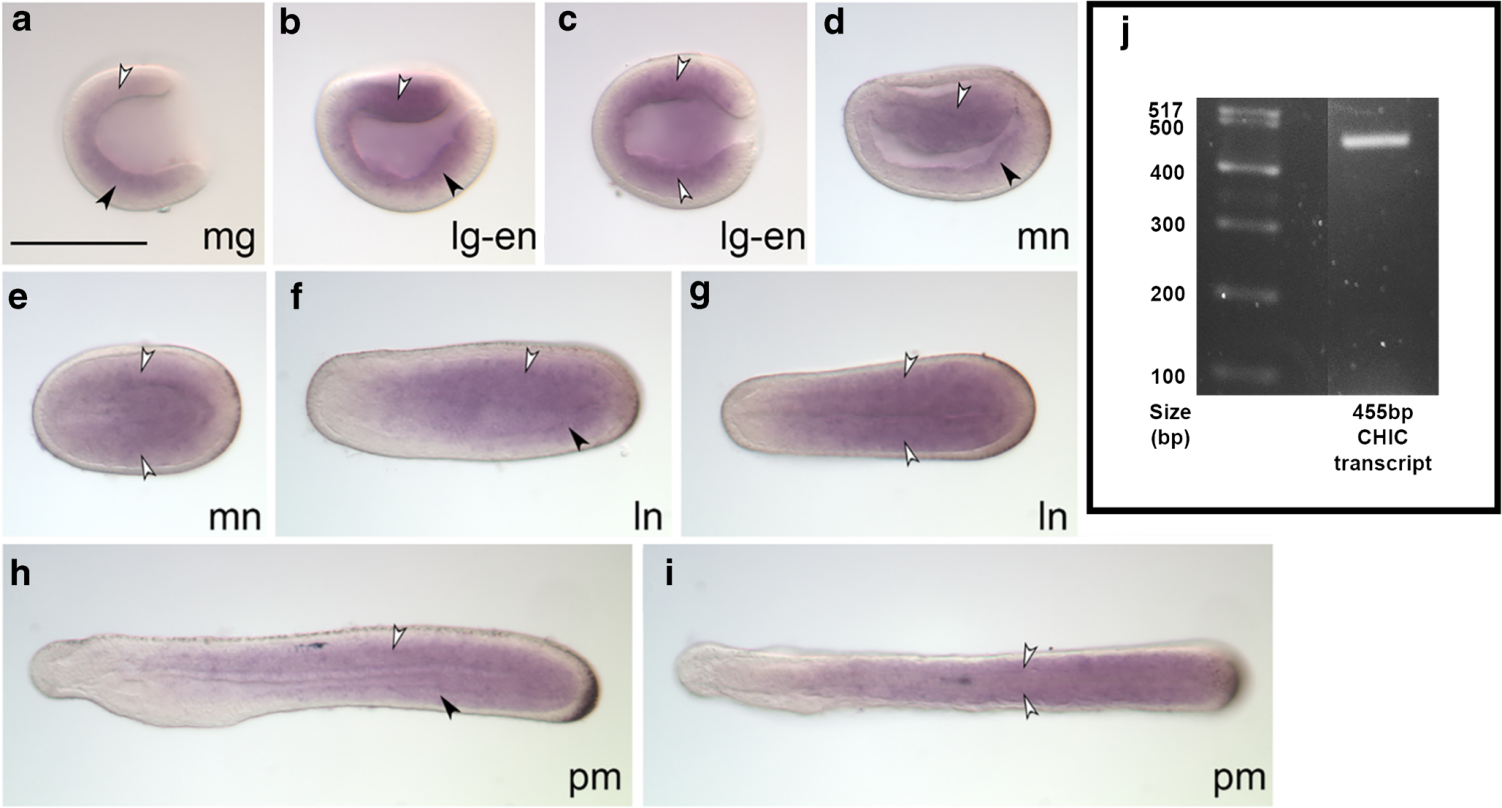
Expression of *B. lanceolatum CHIC* transcripts within embryos and gonadal tissue. **a–i** Embryonic expression of *B. lanceolatum CHIC* is shown from the mid-gastrula (**a**) to the pre-mouth (**i**) stages of development. Expression begins in the ventral endoderm (black arrowheads) and dorsal mesoderm (white arrowheads) at the mid-gastrula stage to the late gastrula (**a**–**c**) but is clearly absent from the ectoderm. This expression continues through the early-late neurula (**d**–**g**), with expression absent from both the ectoderm and neural tube, as well as becoming restricted from the far anterior of the embryo. Throughout the late neurula–pre-mouth stages (**g–i**), expression reaches as far anteriorly as the first somite dorsally and ventrally up to the pharynx but is still notably absent from the neural tube, cerebral vesicle and ectoderm. **a**, **b**, **d**, **f**, **h** represent lateral views, whilst **c**, **e**, **g**, **i** represent dorsal views. **j** shows the 455-bp *AmphiCHIC* mRNA transcript cloned from *B. lanceolatum* gonadal total mRNA that was used to create an antisense RNA hybridisation probe. mg mid-gastrula, lg late gastrula, en early neurula, mn mid-neurula, ln late neurula, pm pre-mouth. Scale bar represents 100 μm

As both *AmphiCHIC* and *AmphiSYCP1* appear to share the same bidirectional promoter, it is possible that the two would share some similarities in their expression. In order to examine this, the expression of *AmphiCHIC* was assayed by both in situ hybridisation and RT-PCR in the same stages and tissues examined for *AmphiSYCP1* (Fig. 5a–i).

In the mid-gastrula, *AmphiCHIC* expression can be seen throughout the mesendoderm but is absent from the ectoderm (Fig. 5a). This continues into the late gastrula/early neurula (Fig. 5b, c). In the mid-neurula, *AmphiCHIC* expression appears to be excluded from the anterior and posterior extremes of the embryo (Fig. 5d, e). Whilst the mesoderm expression (white arrowhead) extends almost all the way to the anterior, the ventral endoderm expression is much more restricted to the posterior of the embryo (black arrowhead) (Fig. 5d). In the late neurula, expression is similar to that of the mid-neurula, though expression now extends into the posterior tailbud but is still absent from the ectoderm (Fig. 5f, g). In this stage, the lack of anterior expression is now more noticable, and though the dorsal mesoderm expression (white arrowhead) extends further anteriorly than the ventral endoderm expression (black arrowhead), the far anterior portion of the embryo is clearly lacking any *AmphiCHIC* expression (Fig. 5f, g). This expression pattern continues to the pre-mouth stage, with expression only reaching as far anteriorly as the presumptive pharynx along the ventral side and into the first somite dorsally (Fig. 5h, i). In concurrence with *AmphiSYCP1* gonadal expression, a 455-bp spliced *AmphiCHIC* transcript, spanning *AmphiCHIC* exon 1 to exon 6, was amplified and cloned from adult *B. lanceolatum* gonadal cDNA (Fig. 5j).

### *SYCP1* is widely conserved across the Metazoa

**Fig. 6:**
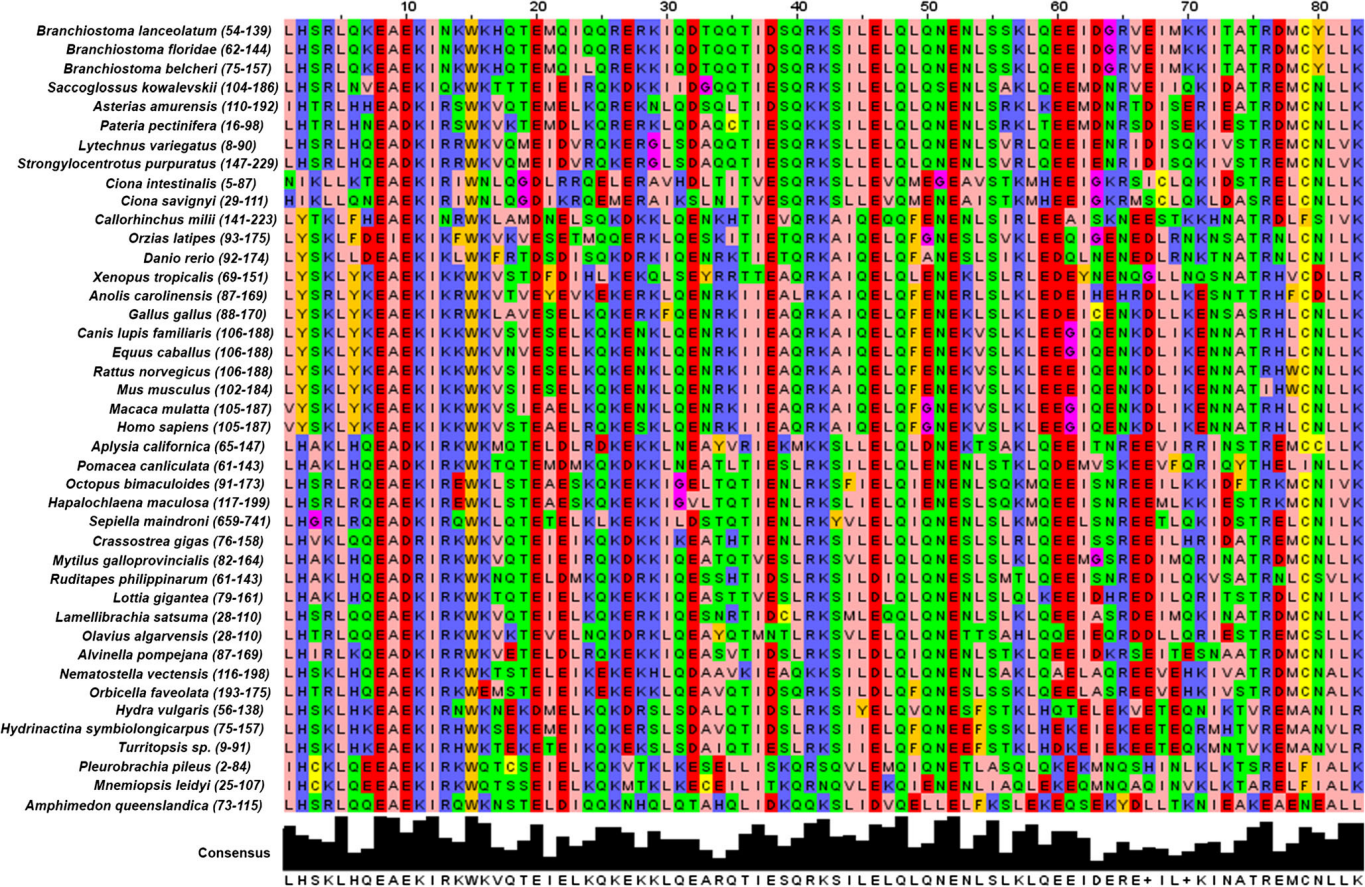
The CM1 motif of SYCP1 is highly conserved across the Metazoa. A CLUSTALW protein multiple alignment of the CM1 domains of SYCP1 shows a high level of conservation across an 83-aa motif across the metazoan species examined. Conservation is visualised with false colour using the Zappo colour table for amino acids. Effort made to identify transcripts from phyla underrepresented within (Fraune et al. 2012a). A consensus sequence made up of the most abundant amino acid for each position is given in black. The names of species used are given to the left of the alignment, and species are organised roughly according to the current known phylogeny with amphioxus species as the focus. The numbers in parentheses indicate the position of the CM1 motif amino acids within the obtained native peptide sequence

Fraune et al. (2012a) showed that *SYCP1* was much more highly conserved across the metazoans than previously thought, greatly extending the evolutionary history of this gene. With the ever-growing list of genome sequences available, greater taxon sampling can now be achieved to further address *SYCP1* evolutionary history. With this aim, a multiple alignment of SYCP1 proteins was produced, highlighting the conserved CM1 domain identified by Fraune et al. (2012a) and aiming to better sample underrepresented phyla. The chimaera (*Callorhinchus milii*) was added to the Vertebrata as a basal fish lineage, as well as additional echinoderm species, including a second echinoid (*Lytechinus variegatus*) and two members of the asteroids (starfish) (*Asterias amurensis* and *Pateria pectinifera*). Additionally, a single hemichordate SYCP1 sequence from *Saccoglossus kowalevskii* was identified, giving examples of SYCP1 from all three main deuterostome phyla. In the Protostomia, lophotrochozoan sequences were expanded greatly within the Mollusca with the addition of a gastropod (*Pomacea canaliculata*), two bivalves (*Mytilus galloprovincialis* and *Ruditapes philippinarum*) and three cephalopods (*Octopus bimaculoides*, *Hapalochlaena maculosa* and *Sepiella maindroni*). Two additional annelid sequences were also obtained (*Lamellibrachia satsuma* and *Olavius algarvensis*). No additional ecdysozoan members were obtained (beyond the highly divergent and short *Petrolisthes cinctipes* sequence fragment found by Fraune et al. 2012a). Several additional members of the Cnidaria were obtained beyond *Hydra vulgaris* (*Orbicella faveolata*, *Hydractina symbiolongicarpus* and *Turritopsis sp*.), and a full length *Nematostella vectensis SYCP1* sequence was obtained to replace the short EST read previously used by Fraune et al. (2012a). The Ctenophora was expanded to include the sea walnut *Mnemiopsis leidyi* as well as *Pleurobrachia pileus.* Finally, the poriferan *Amphimedon queenslandica* represents the sole example of SYCP1 so far identified in this phylum. Full species names, groups and accession numbers are given in Supplementary file 4. Whilst a full SYCP1 protein alignment can be found in Fig. S1, the CM1 conserved motif (see the “Materials and methods” section) provides much better resolution for distinguishing SYCP1 sequences from other coiled-coil proteins. Figure 6 illustrates the high level of conservation of the CM1 motif across the Metazoa.

**Fig. 7:**
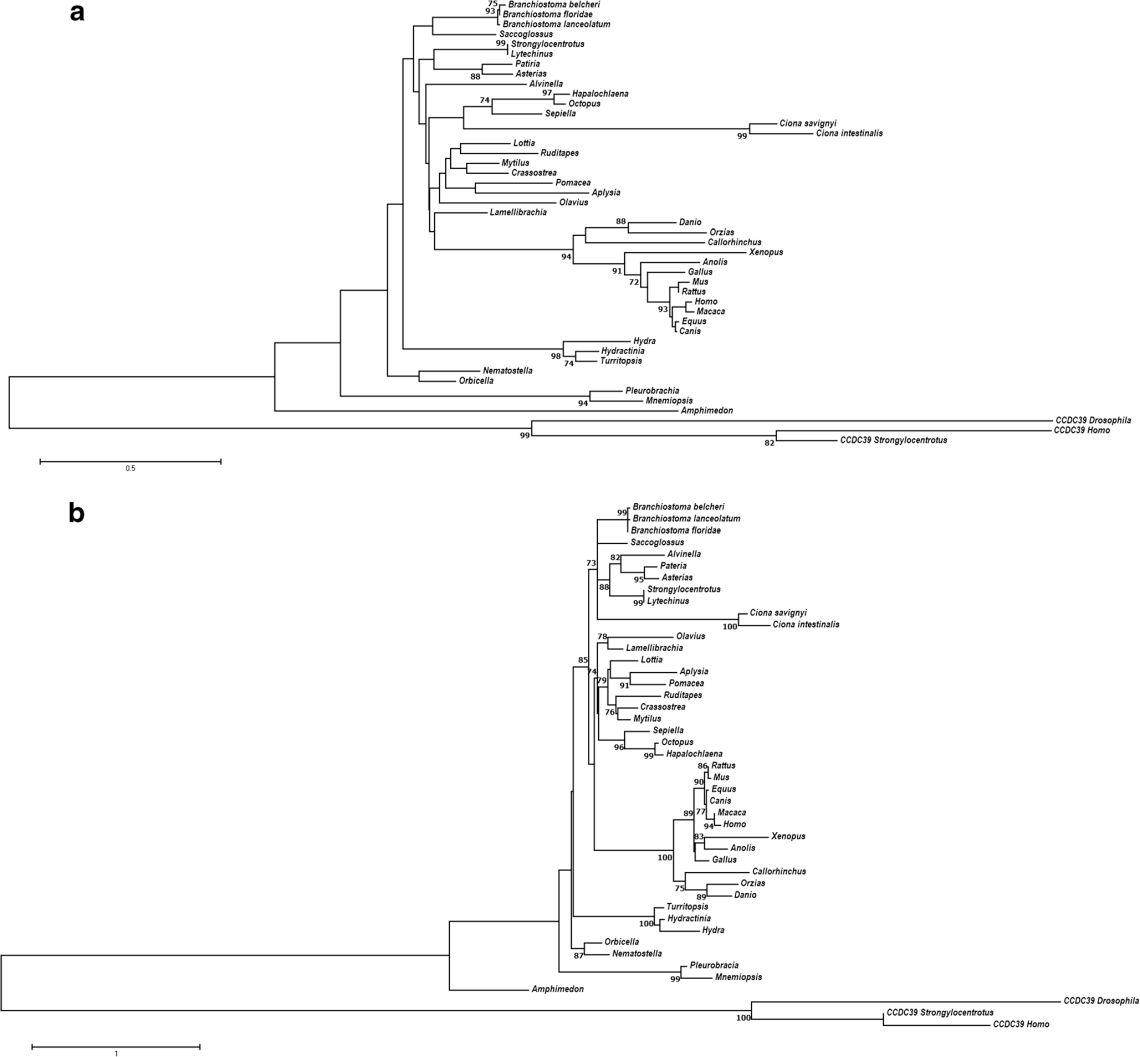
Phylogeny of metazoan SYCP1 CM1 motifs. **a** Neighbour-joining tree built using the 83-aa CM1 domain of SYCP1 proteins, using the JTT + G matrix with 1000 bootstraps, a gamma shape parameter of 2.157 and a 95% partial-deletion cutoff. **b** Maximum-likelihood tree built using the 83-aa CM1 domain of SYCP1 proteins, using the LG + G model with four discrete gamma categories, using all sites, and branch support calculated using the aLRT SH-like statistic (Anisimova and Gascuel 2006), with bootstrap support values provided as a function of the aLRT statistic. CCDC39 proteins were used as an outgroup to SYCP1. Bootstrap values over 70% are given. Longer branch lengths equate to a further evolutionary distance between nodes. NJ trees were built using MEGA7, whilst ML trees were built using PHYML (see the “Materials and methods” section). The analysis involved a total of 45 amino acid sequences

To deduce possible evolutionary relationships between these SYCP1 sequences, phylogenetic trees were produced for the SYCP1 CM1 domain (see the “Materials and methods” section). Bootstrap support values are low on many branches, for both NJ (Fig. 7a) and ML (Fig. 7b) analyses. Nevertheless, the vertebrates group together with significant support, as do the different vertebrate groups such as mammals and fish. The remainder of the phylogeny grouped roughly as expected according to known species relationships, albeit with very low support. The paucity of significant node support values is likely due to the short length of the CM1 motif. Several smaller clades do, however, show consistently high support, often where better representation of more closely related species can be found. These include the Branchiostomidae, Asteroidea, Echinoidia, Cephalopoda, Tunicata, Hydrozoa and Ctenophora. The Cephalopoda and Tunicata are often grouped together, likely due to long-branch attraction. *Alvinella* also often groups with the Asteroidea rather than the Lophotrochozoa, in this case due to several key amino acid similarities at positions 65–70 seen in Fig. 6. Whether this convergent similarity has any functional significance remains to be seen.

## Discussion

### Amphioxus *SYCP1* is a transcribed retrogene that replaced its parental multi-exonic copy before the divergence of the *Branchiostoma* genus

Comparisons between the three amphioxus genomes show that amphioxus *SYCP1* is present as a single coding exon within *B. floridae*, *B. lanceolatum* and *B. belcheri* (Fig. 1). Thus, we can conclude that an *SYCP1* retrogene must have been present upstream of the ParaHox cluster, between *CHIC* and *Gsx*, before the divergence of these three species. It will be necessary to examine the ParaHox cluster of both *Asymmetron* (Yue et al. 2014) and *Epigonichthys* (Nohara et al. 2005), as the only two other amphioxus groups known besides *Branchiostoma* species, to determine if this instance of retrogene replacement is typical for all amphioxus.

The presence of multi-exonic *SYCP1* genes throughout the rest of the Bilateria, within the vertebrates, echinoderms and Lophotrochozoa (Table S1), makes it highly likely that both amphioxus and *Ciona intestinalis SYCP1* genes evolved via retrotransposition and replaced a multi-exonic ancestral parent gene. Indeed, in much the same manner as *AmphiSYCP1*, there is only one single-exon copy of *SYCP1* within the *Ciona*, though it has inserted into a different locus and does not lie next to any of the ParaHox genes (Table S1**)**. Retrogene replacement appears to be a relatively common mechanism in *Ciona* (Kim et al. 2014), and with the genome compaction, dispersal and gene loss in tunicates (Berna and Alvarez-Valin 2014; Dehal et al. 2002; Hughes and Friedman 2005), this mechanism may contribute to their fast genome evolution. In addition, the existence of multiple instances of *SYCP1* retrogene copies within the mouse (Sage et al. 1997) also suggests that *SYCP1* is perhaps prone to retrotransposition, at least within the chordates. The expression of *SYCP1* within the germ line may very well make *SYCP1* a target for the ‘out-of-the-testis’ route of retrogene production (Kleene et al. 1998; Vinckenbosch et al. 2006) and eventual replacement of the parent gene by the retrocopy (Ciomborowska et al. 2013).

### Amphioxus *SYCP1* has evolved a de novo multi-exonic 5′ UTR that may originate from a co-opted bidirectional *CHIC* promoter

Our RT-PCR data, combined with transcriptome and genome sequence data, indicates the presence of a multi-exonic 5′ UTR stretching upstream from the *SYCP1* coding sequence between *SYCP1* and *CHIC* (Fig. 4). Promoter analysis revealed no promoter present immediately upstream of the *SYCP1* coding region; however, it did reveal a putative promoter lying upstream of *CHIC* exon 1 (Fig. 4). This putative promoter was identified with high support values in all three of the programs used for prediction (NNPP, TSSW and ProScan 1.7). These three programs were used in order to provide multiple alternative methods of both identification and support for putative promoter sequences (Prestridge 1995; Reese 2001; Solovyev et al. 2006; Solovyev et al. 2010) and mitigate against any shortcomings of each program. This approach leads to the prediction of one promoter region that is common to all three approaches, making it much more likely that this site is indeed a bona fide promoter sequence. To corroborate this, the same analysis was carried out upon both *B. floridae* and *B. lanceolatum*, with both species producing very similar results despite the high levels of polymorphism that exist in non-coding sequence in amphioxus species (Huang et al. 2012).

Intriguingly, promoter predictions across both species indicate a promoter on both positive and negative strands at this site, raising the possibility that this may be a bidirectional promoter. The presence of this promoter overlapping the first exons of both *CHIC* and *SYCP1* 5′ UTR is certainly consistent with this (Fig. 4). This raises the possibility of an interesting evolutionary scenario, in which *AmphiSYCP1* has co-opted a *CHIC* promoter, whilst retaining its essential germline expression. *SYCP1* would then have either evolved its own de novo 5′ UTR in order to take advantage of this bidirectional promoter or co-opted UTR sequence from the adjacent *CHIC* gene. It is likely that the orientation of the two genes, as well as the position of the predicted promoter sequence, precludes the co-option of 5′ UTR elements from *CHIC*. Also, we find no evidence for, but cannot conclusively exclude, a third possibility that amphioxus *SYCP1* inserted into an intervening gene between *CHIC* and *Gsx*, possibly replacing all of this gene except for these few 5′ non-coding exons such that *AmphiSYCP1* inherited these non-coding exons from this additional, but now absent, gene. Whilst it may seem a large evolutionary leap for a retrogene to evolve a 5′ UTR or co-opt an existing nearby regulatory element, this has been seen to occur with other bilaterian retrogenes. For example, a genome-wide screen of retrogenes within *Drosophila* revealed that several regulatory motifs were overrepresented in the cis-regulatory elements of testis-expressed retrogenes and that specific regulatory motifs had been selectively recruited by retrogenes from their new genomic loci (Bai et al. 2009). Indeed, it seems that retrogenes rarely bring along any active regulatory elements of their own when inserting into their new locus (Bai et al. 2008). Another key study selectively looked at the evolution of introns within retrogenes of mammals and found that most introns found associated with retrogenes occurred in the 5′ flanking sequence to the retrogene insertion site, which is linked to the recruitment of distal promoters (Fablet et al. 2009). There may even be selective pressure for the evolution of multi-exonic 5′ UTRs within retrogenes, as those with introns display higher transcription levels and broader expression patterns than those without. Fablet et al. (2009) propose a scenario where 5′ exon-intron structures evolve de novo or through fusion to the 5′ UTR of a neighbouring gene as a direct link to the recruitment of a distant promoter by a retrogene. It is also noteworthy that of those recruited by distant promoters and that gained 5′ exon-intron UTR structures, most were recruited by bidirectional CpG promoters (Fablet et al., 2009). It is becoming clear that the phenomena of retrogenes recruiting regulatory elements from regions flanking their insert site, as well as retrogenes gaining introns, may not be as rare as they once seemed (Kang et al., 2012; Sorourian et al., 2014). There is an abundance of general transcription occurring within cells to which no functional role can be attributed, and lots of non-coding, non-functional RNA is produced (Struhl 2007). It is entirely possible that retrogenes could be co-opting the sequences involved in this pervasive transcription to facilitate their own transcription as part of retrogene evolution.

The combination of 5′ UTR transcript, precise placement of a predicted promoter (perhaps bidirectional) adjacent to both *CHIC* exon 1 and *SYCP1* 5′ UTR exon 1 and broad *CHIC-*like somatic expression of *AmphiSYCP1* in embryos are all consistent with recruitment of a bidirectional *CHIC* promoter by the *AmphiSYCP1* retrogene. *SYCP1* would then have evolved a de novo 5′ intron-exon structure to make use of the distant promoter. A preliminary check for CpG islands within the *CHIC-SYCP1* 5’ UTR region yielded no results, but the identified promoter region could nonetheless still display bidirectionality. Indeed, it is now thought that bidirectionality is an inherent feature of promoters (Wei et al. 2011). Further work could examine this promoter region in a reporter background to test both its bidirectionality and its similarity to *AmphiSYCP1* expression.

### Expression of *AmphiSYCP1* is much broader than expected for a meiosis gene

It is clear from the in situ hybridisation of *AmphiSYCP1* that expression is by no means limited to the germ cells, and typical germ cell markers such as *nanos* and *vasa* show markedly different embryonic expression patterns to *SYCP1* (Dailey et al. 2016; Wu et al. 2011). As *SYCP1* expression is limited to meiotic cells in both vertebrates (Casey et al. 2015; de Vries et al. 2005; Iwai et al. 2006), including primordial germ cells (Zheng et al. 2009), and *Hydra* (Fraune et al. 2012a), it was expected that no embryonic expression would be observed, as the testis and ovaries have not yet formed in amphioxus, or that *SYCP1* would display *nanos*-/*vasa*-like germ cell expression (Wu et al. 2011). Furthermore, if *AmphiSYCP1* had transposed along with its own regulatory elements, such as a promoter region, it might even be expected that germ cell expression is the most likely outcome, as previous work has shown the zebrafish *SYCP1* promoter region to be sufficient to drive GFP transgenes within germ cells (Gautier et al. 2013).

*AmphiSYCP1* is expressed in the endoderm and mesoderm in a broad pattern throughout these tissues and also seems to exhibit spatio-temporal changes in expression. *AmphiSYCP1* is notably absent not only from the ectoderm and the posterior tailbud, but also from the extreme anterior in all stages (Fig. 2). This expression pattern, which is much broader than expected for *SYCP1*, suggests that *AmphiSYCP1* has co-opted regulatory elements from its new genomic locus. It does not appear to have come under the influence of ParaHox regulatory elements, however, as the broad expression pattern observed is not reminiscent of ParaHox expression, and there is no CNS expression, a hallmark of ParaHox genes (Brooke et al. 1998; Osborne et al. 2009). In addition, *AmphiSYCP1* does not exhibit any of the patterns of collinear expression, either spatial or temporal, expected if it had co-opted pan-cluster regulatory elements from the adjacent amphioxus ParaHox cluster. It may, however, have gained some of this somatic expression from its co-option of regulatory elements from the neighbouring *AmphiCHIC* gene.

Our study provides the first description of *AmphiCHIC* expression, and though it is not identical to that of *AmphiSYCP1*, certain similarities can be observed. These are particularly evident in the early stages of development (Figs. 2a–f and 5a–d), where expression is limited to the mesoderm and endoderm and excluded from the ectoderm and neural tube. As embryogenesis progresses, differences in expression become more apparent after closure of the blastopore, though many similarities remain. Expression becomes broader throughout the mesoderm and the endoderm for both *AmphiSYCP1* and *AmphiCHIC* whilst still remaining absent from the ectoderm and neural tube in each case (Figs. 2f–i and 5f–i).

The expressions of *AmphiSYCP1* and *AmphiCHIC* are difficult to compare within other chordate phyla, as neither have been examined in an embryonic spatio-temporal context, with *SYCP1* somatic expression not yet observed at all within the vertebrates. Very little expression data exists even for the vertebrate *CHIC* genes. However, *CHIC1* and *CHIC2* were both originally identified as *Brain x-linked protein* (*Brx*) and *BrX-like translocated in leukaemia* (*BTL*), respectively, and their roles in the regulation of nuclear hormone receptors (Kino et al. 2006) and exocytosis (Cools et al. 2001) have been described. Though the expression of vertebrate CHIC genes was first identified in the brain, both *CHIC1* and *CHIC2* also exhibit expression in the testis, ovary, uterus, endomesoderm, intestine, ectoderm, many secretary organs of the digestive tract, thyroid, prostate and pineal gland (data from http://www.proteinatlas.org/ (Uhlén et al. 2015)). CHIC genes seem to show expression in a range of tissues, many of which have secretory functions. This may be linked to the described role in plasma membranes and vesicles and exocytosis (Cools et al. 2001). This expression also holds true for the protostome CHIC homologues *TAG-266* (*Caenorhabditis elegans*) (Consortium CeS 1998) and *CG5938* (*Drosophila melanogaster*) (Hoskins et al. 2015). Since bilaterian CHIC genes are expressed in the testis and ovaries, co-option of *CHIC* regulatory elements would still allow *AmphiSYCP1* to carry out its meiotic function and also give the potential to evolve new expression domains within somatic tissues.

One other example of bilaterian *SYCP1* expression is particularly noteworthy with respect to the expression of *AmphiSYCP1* within the embryonic somatic tissue. In the sea urchin *Strongylocentrotus purpuratus*, *SYCP1* is found to be expressed in the larvae throughout the adult rudiment (Yajima et al. 2013). This structure goes on to form most of the adult animal, and the larvae is largely cast off or reabsorbed. Determining the function of sea urchin *SYCP1*, along with other meiotic genes that are expressed throughout the adult rudiment, awaits further research. It remains to be seen whether the embryonic expression of meiotic genes is a more widespread phenomenon, or indeed whether *SYCP1* carries out a yet unknown function within embryogenesis or somatic cells. It is possible, however, that transcription of *SYCP1* is not indicative of any function in somatic cells. Mammalian studies have indicated that meiotic genes can be activated in initially broad domains and only later become restricted to germ cells (Saitou et al. 2002; Saitou et al. 2003), with transcription often beginning prior to the initiation of meiotic events (Kimble and Page 2007). As such, it is entirely possible that the somatic expression of *AmphiSYCP1* transcripts merely represents non-functional transcription. It is also possible that *SYCP1* transcription is allowed to proceed in somatic tissues as it has no negative effect or that the improvement to transcription in target tissues granted by co-opted regulatory elements outweighs any transcriptional costs in somatic tissues.

### *SYCP1* is widely conserved across the Metazoa, except for its absence from the Ecdysozoa

As Fraune et al. (2012a) showed, SYCP1 proteins are much more deeply conserved across the Metazoa than previously believed, along with several other components of the synaptonemal complexes, suggesting deep conservation of meiotic machinery (Fraune et al. 2012a; Fraune et al. 2013; Fraune et al. 2012b; Fraune et al. 2014). This work on both *Hydra* (Fraune et al. 2012a; Fraune et al. 2013; Fraune et al. 2012b; Fraune et al. 2014) and sea urchin (Yajima et al., 2013) synaptonemal complex proteins has not only identified these genes, but also confirmed their expression. The phylogenetic study carried out here has sought to extend the work of Fraune et al. (2012a), identifying *SYCP1* genes and proteins throughout the Metazoa, by utilising the wealth of new genome sequences that have become available. This has allowed a broader sampling of *SYCP1* from within the non-chordate deuterostomes, specifically, with the addition of an echinoid, two asteroids and one hemichordate sequence from the Ambulacraria, providing at least one example of *SYCP1* from each deuterostome phylum, as well as much greater representation within both the Lophotrochozoa and Cnidaria.

The Ecdysozoa are notably absent from the list of *SYCP1*-possessing taxa. Fraune et al. (2012a) included a *Petrolisthes cinctipes* sequence as the sole ecdysozoan representative. This sequence was included in initial phylogenetic analysis, but consistently groups basal to all lineages other than *Pleurobrachia* and *Amphimedon*, including the Cnidaria. Further examination of this sequence fragment shows it to be both short and highly divergent even in comparison to cnidarian, poriferan and ctenophore sequences. Indeed, when included in phylogenies, this sequence proved to be unstable, and iterations of the alignment carried out with CLUSTALW (Larkin et al. 2007) and MUSCLE (Edgar 2004) did not align the *Petrolisthes* ESTs to the conserved CM1 domain at all. Instead, this crustacean sequence aligned further towards the coiled-coil containing-C terminus of other SYCP1 proteins.

To attempt to validate this sequence as a bona fide SYCP1, we searched for *SYCP1* from other ecdysozoan groups, including more basal arthropod lineages such as the myriapod *Strigamia maritima* (Chipman et al. 2014) and spiders (Sanggaard et al. 2014). This search provided no *SYCP1* candidates. Multiple peptide sequences, including mouse, amphioxus, *Hydra* and *Amphimedon* SYCP1 sequences, were all used as queries when looking for ecdysozoan sequences, as well as BLAST searches using only the conserved CM1 domains. This is even more relevant in light of the lineage-specific components of synaptonemal complexes of well-studied ecdysozoans such as *Drosophila melanogaster* and *Caenorhabditis elegans*, both species having independently evolved functionally similar, but novel, synaptonemal complex proteins that fulfil the same functional role as SYCP1 in other metazoans, (Bogdanov I et al. 2002; Bogdanov et al. 2003; Colaiácovo et al. 2003; MacQueen et al. 2002; Page and Hawley 2001; Schild-Prufert et al. 2011; Smolikov et al. 2007). The complete lack of SYCP1 proteins in any other ecdysozoan and evolution of lineage-specific synaptonemal proteins in both *D. melanogaster* and *C. elegans* suggest that the *Petrolisthes* sequence could be a case of misidentification or contamination. It is also possible that the ‘SYCP1’ hits are not, in fact, SYCP1 and that a longer sequence would reveal a lack of homology. This sequence could also simply be an instance of another coiled-coil protein, of which there are many, with the small sequence preventing proper identification. The precise point in animal evolution at which the transition was made from the typical metazoan SYCP1 system to the ecdysozoan alternatives remains to be resolved.

## Conclusion

In this study, the amphioxus retrogene *AmphiSYCP1* has been characterised, highlighting its expression and regulation in relation to the surrounding genomic locus into which it has inserted. In situ hybridisation of *AmphiSYCP1* revealed widespread embryonic and somatic expression unexpected for a meiotic gene, whilst promoter and transcriptional analyses reveal that *AmphiSYCP1* seems to have not only co-opted a bidirectional promoter from the adjacent gene *AmphiCHIC*, but also evolved a de novo multi-exonic 5′ UTR in order to make use of this promoter. The conservation of this regulatory structure between *B. lanceolatum* and *B. floridae*, as well as the presence of two different *AmphiSYCP1* isoforms with differing 5′ UTR exon structures, implies an important role for this 5′ UTR structure in the regulation and expression of *AmphiSYCP1*. We also describe the expression of the adjacent gene *AmphiCHIC*. This supports the hypothesis that *AmphiSYCP1* has co-opted a bidirectional *AmphiCHIC* promoter, with *AmphiCHIC* displaying a similar expression pattern to that of *AmphiSYCP1* during embryonic development. *AmphiSYCP1* does not appear to have co-opted regulatory patterns from the adjacent ParaHox cluster, however, despite its proximity to *AmphiGsx*. Finally, phylogenetic analysis of *SYCP1* proteins from across the Metazoa supports the ancient origin of *SYCP1* even though resolution is poor outside of the Vertebrata, but in contrast to Fraune et al. (2012a), we conclude that *SYCP1* has been lost within the Ecdysozoa.

## Materials and methods

### Origin and culture of *B. lanceolatum* individuals

Live adult *B. lanceolatum* were collected by the Plymouth Marine Laboratory, UK, and were transferred in 2011 to the aquarium system of the Gatty Marine Laboratory at the University of St. Andrews, UK, where they were kept in culture with continual aeration and circulating ambient-temperature seawater under a 16:8-h (light/dark) photoperiod until harvested. Animals were fed once or twice a day with a mixed diet of unicellular red algae *Rhinomonas reticulata* supplemented with MarineSnow (Two Little Fishies, Inc), a planktonic solution for filter-feeding marine invertebrates. Gravid animals used for gonadal RNA extraction were fixed directly in RNAlater for 24 h, and the gonads were then dissected. Embryos were collected by spawning of ripe amphioxus at the facilities of Laboratoire Aragó in the summer of 2010. These were induced by heat stimulation as described in Fuentes et al. (2007), and embryonic stages (gastrula, early neurula, mid-neurula, late neurula and early larval stages) were collected at regular intervals and fixed in 4% (*m*/*v*) paraformaldehyde in MOPS buffer for 1 h at room temperature or overnight at 4 °C and then transferred into 70% ethanol and stored at − 20 °C until use (Holland et al., 1996). Embryos of mid-late neurula stages were kindly gifted by Dr. Ildiko Somorjai (University of St. Andrews).

### Isolation of adult *B. lanceolatum* gonadal cDNA

Ripe gonads were dissected from a single gravid adult *B. lanceolatum* individual stored in RNAlater (Sigma). This was then rinsed in RNase-free water (Fisher Scientific) several times before being transferred to 1 ml TriReagent (Sigma) on ice. The tissue was homogenised in a D-Matrix tube (MP Biomedicals) in a Fastprep FP120 cell homogeniser (Thermo Savant) at 6 m/s for 40 s. Phenol/chloroform extractions were carried out until no denatured protein material could be observed at the aqueous/chloroform interface. The aqueous phase was then taken and precipitated with an equal volume of isopropanol, followed by a 70% ethanol wash. The dry RNA pellet was then resuspended in RNase-free water and stored at − 80 °C for long-term storage. An aliquot was stored at − 20 °C for immediate use. Due to the lack of introns within the *AmphiSYCP1* coding region, an additional DNase I treatment was carried out upon the RNA to ensure the removal of any possible genomic DNA contamination. One microlitre of DNase I (Fermentas) was added to an aliquot of the RNA solution and incubated at 37 °C for 30 min. One microlitre of 50 mM EDTA was then added to this, and the sample was heat-deactivated at 65 °C for 10 min. Pure, uncontaminated RNA was then repurified using the Isolate RNA mini kit (Bioline) according to the manufacturer’s instructions. cDNA was produced from this purified adult *B. lanceolatum* gonadal RNA sample using the Tetro cDNA synthesis kit (Bioline) following the manufacturer’s instructions, using oligo(dT)s to prime the reaction.

### Cloning of *SYCP1* and *CHIC* transcripts

*B. lanceolatum SYCP1* coding sequence, *SYCP1* 5′ UTR and *CHIC* transcripts were obtained by PCR using BIOTAQ polymerase (Bioline) from adult *B. lanceolatum* gonadal cDNA preparations. PCRs were set up with a total volume of 50 μl in a 0.2-ml PCR tube. All reactions used 5 μl 10 × NH_4_ buffer, 2 μl 50 mM MgCl_2_ solution, 2 μl (5 μl for *AmphiSYCP1*) 10 mM dNTPs, 1 μl 20 μM forward primer, 1 μl 20 μM reverse primer, 1 μl of a one-tenth dilution of adult *B. lanceolatum* gonadal cDNA, 0.5‐1 μl 5 U/μl BIOTAQ and ddH_2_O up to a total volume of 50 μl. Primer sequences, annealing temperatures and elongation times used were as follows: AmphiSYCP1 F (**GCAGGTGTRTYATCAGCAAGAG**) and AmphiSYCP1 R (**ACTCRAAGAAGCCAAAAACAGT**) at 56 °C annealing temperature with 3-min extension time, B.la_SYCP15’UTR_v2 F (**AGAGAGGAGGAACAGAGGGATTTT**) and B.la_SYCP15′UTR_v2 R (**CCTCAACATTAGCAGCATGATCTTT**) at 58 °C annealing temperature with 45-s extension time, B.la_CHIC_ex1 F (**GAGCGGCTTATGGAGGAACA**) and B.la_CHIC_ex6 R (**AGTCTGGTCTGTGGATGGGA**) at 60 °C with 45-s extension time. PCR products for *B. lanceolatum SYCP1* coding (3121 bp), *SYCP1* 5′ UTR (307 and 246 bp) and *CHIC* (455 bp) were then gel-purified and cloned into pGEM-T Easy according to the manufacturer’s instructions. These clones were then sequenced in both forward and reverse orientations using 3.2 μM T7 and SP6 primers, with the additional 3.2 μM B.la SYCP1-centre F (**AGTCTCTTCAAGATCAGCTGCAA**) and B.la SYCP1-centre R (**CTTTATCTTCGATGGTTTTCTTCA**) primers used to sequence the centre of the large 3121-bp SYCP1 coding product. Accession numbers for cloned sequences are provided in the methods below.

### In situ hybridisation

PCR templates were synthesised from the *B. lanceolatum SYCP1* coding region (3121 bp), *SYCP1* 5′ UTR (307 bp) and *CHIC* (455 bp) pGEM-T Easy clones using M13 primers, and DIG labelled antisense RNA probes then synthesised from these templates using T7 polymerase. The large *SYCP1* coding antisense probe underwent an additional partial alkaline hydrolysis step by adding 30 μl 200 mM Na_2_CO_3_, 20 μl 200 mM NaHCO_3_ and RNAse-free water up to a total volume of 100 μl followed by incubation for 15 min at 60 °C. Antisense RNA probes were purified using mini quick-spin columns (Roche) according to the manufacturer’s protocol. In situ hybridisation was carried out upon gastrula to pre-mouth stage *B. lanceolatum* embryos according to Holland et al. (1996) with the following modifications. Amphioxus embryos were rehydrated through an ethanol series into PBT and then digested for 5 min at room temperature in 2 μg/ml proteinase K, except for pre-mouth embryos and 2-day larvae which were proteinase K-treated for 10 min. After triethanolamine/acetic anhydride washes, embryos were washed once in PBT for 1-min with rotation, then again in PBT for 5-min with rotation. This was then changed for 100 μl of hybridisation buffer pre-warmed to 60 °C and rotated for 1 min. This was changed for fresh hybridisation buffer and rocked for 2 h. Antisense RNA probe was mixed in 1/50 dilutions in fresh warm hybridisation buffer and denatured at 70 °C for 10 min, before being added to the embryos. These were then rocked overnight at either 60 or 62 °C. RNase steps were carried out with 2 μl 10 mg/ml RNaseA and 1 μl RNaseT1 (10,000 U/ml) in 1 ml of wash solution 3, and 250 μl was added per well. After wash solution 5, 200 μl of blocking solution was added to the embryos and rotated for 3 h at room temperature. Blocking solution was replaced with 1:2000 antidigoxigenin-alkaline phosphatase (Alkaline Phosphatase) Fab fragments in blocking solution and incubated overnight at 4 °C. Embryos were washed four times NaPBT for 20 min each at room temperature, before three washes in AP− followed by three washes in AP+. AP+ was exchanged for staining buffer, and embryos were left in the dark at room temperature for the colour to develop. The final post-staining procedure consisted of three washes in AP− for 10 min each, rotating in the dark, followed by three washes in NaPBT for 10 min each, rotating in the dark. Embryos were finally fixed in 4% PFA in NaPBS for 1 h at room temperature, washed twice in NaPBT for 10 min each and transferred to 80% glycerol to clear.

### Bioinformatic prediction of candidate *SYCP1* promoters

In order to utilise a more robust approach to promoter prediction, three independent promoter prediction programs utilising different prediction algorithms were employed: NNPP (Reese et al. 1996; Reese and Eeckman 1995), TSSW (Solovyev et al. 2010) and WWW Promoter Scan (Prestridge 1995), which uses ProScan 1.7. Default settings were used for all three prediction software programs.

### Analysis of SYCP1 conservation

The position of the retrogene *AmphiSYCP1* adjacent to the *B. floridae* ParaHox cluster was confirmed by TBLASTN search against the *B. floridae* genome, using the *M. musculus SYCP1* peptide sequence as a query sequence, and also through a BLASTN search using the previously identified *AmphiSYCP1* nucleotide sequence from the *B. floridae* ParaHox PACs 33B4 and 36D2 (Ferrier et al. 2005). The resulting *B. floridae SYCP1* nucleotide and peptide sequences were then used as a query to perform both BLASTN and TBLASTN searches against the *B. lanceolatum* (*B. lanceolatum* genome consortium, unpublished) and *B. belcheri* (Huang et al. 2014) genomes to confirm the presence of the *AmphiSYCP1* retrogene adjacent to the *B. lanceolatum* and *B. belcheri* ParaHox clusters. *B. floridae SYCP1* 5′ and 3′ EST reads were obtained through BLASTN searches against the NCBI EST database using the *B. floridae SYCP1* nucleotide sequence.

SYCP1 protein sequences were acquired by either TBLASTN or BLASTP searches using the *B. floridae SYCP1*, *M. musculus SYCP1* or *Hydra SYCP1* peptide sequences as a query against protein, transcriptomic shotgun assembly, whole-genome shotgun assembly and EST databases using NCBI, UNIPROT and JGI databases. Sequences were then aligned using CLUSTAL Omega (Sievers et al. 2011) within Jalview (Waterhouse et al. 2009), using the default settings. An 83-amino acid (aa) ‘CM1’ conserved domain, identified within (Fraune et al. 2012a), was extracted and used to determine evolutionary relationships. The analysis involved 45 amino acid sequences in total. ProtTest3.2 (Abascal et al. 2005) and PHYML (Guindon et al. 2010) were used to infer the best-fit model for building phylogenetic trees. Neighbour-joining and maximum-likelihood trees were determined using MEGA7 (Kumar et al. 2016) and PHYML (Guindon et al. 2010), respectively. A neighbour-joining tree was built using the JTT + G model with 1000 bootstraps, a gamma shape parameter of 2.157 and a 95% partial-deletion cutoff. A maximum-likelihood tree was built using the LG + G model with four discrete gamma categories, using all sites, and branch support calculated using the aLRT SH-like statistic (Anisimova and Gascuel 2006), with bootstrap support values provided as a function of the aLRT statistic. CDCC39 sequences from human, sea urchin and fruit fly were obtained and used as an outgroup to help root the phylogenetic trees. This outgroup was chosen as a related coiled-coil domain protein and to maintain comparison with the results of Fraune et al. (2012a).

### Accession numbers

GenBank accession numbers for sequences cloned within this study are as follows:

*B. lanceolatum SYCP1* 5′UTR + coding region isoforms: [SYCP1_3Exon_5primeUTR_Isoform_mRNA: **MF076789**, SYCP1_2Exon_5primeUTR_Isoform_mRNA: **MF076790**].

*B. lanceolatum CHIC* mRNA fragment: [Blan_CHIC_gonadal_mRNA: **MF399210**].

## Electronic supplementary material

Figure S1: Full-length protein alignment of metazoan SYCP1 proteins. A CLUSTAL W protein multiple alignment of SYCP1 proteins from across the Metazoa. Conservation is visualised with false colour using the Zappo colour table for amino acids. Effort was made to identify transcripts from phyla underrepresented within (Fraune et al. 2012a). A consensus sequence made up of the most abundant amino acid for each position is given in black. Names of species used are given to the left of the alignment and species are organised roughly according to the current known phylogeny with amphioxus species as the focus. Table S1: Metazoan SYCP1 intron length comparison. Intron, exon, and transcribed gene lengths for the *SYCP1* gene of several metazoan species are shown in base pair length (bp). The genome version and scaffold number are given for each species, or accession numbers where sequences were obtained from a clone. Table S2: Amphioxus SYCP1 predicted promoter sequences. Sequences for the predicted promoter sequences seen in figure 4 are given, with bold italics denoting the position of predicted transcriptional start site (TSS) where possible. Table S3: Metazoan SYCP1 sequences. Sequences collected for protein alignment and phylogeny are listed. The species is given with the common name in brackets, along with the phylogenetic grouping of the species. Finally accession numbers for all sequences used are given. (PDF 16315 kb)
