# Supplementary material for: Amphioxus SYCP1: a case of retrogene replacement and co-option of regulatory elements adjacent to the ParaHox cluster
Source: Dev Genes Evol. 2018 Jan 2;228(1):13–30. doi: 10.1007/s00427-017-0600-9 (PMC5803294; doi:10.1007/s00427-017-0600-9)
Supplement: Supplementary file 1 — Figure S1: Full-length protein alignment of metazoan SYCP1 proteins. A CLUSTAL W protein multiple alignment of SYCP1 proteins from across the Metazoa. Conservation is visualised with false colour using the Zappo colour table for amino acids. Effort was made to identify transcripts from phyla underrepresented within (Fraune et al. 2012a). A consensus sequence made up of the most abundant amino acid for each position is given in black. Names of species used are given to the left of the alignment and species are organised roughly according to the current known phylogeny with amphioxus species as the focus. Table S1: Metazoan SYCP1 intron length comparison. Intron, exon, and transcribed gene lengths for the SYCP1 gene of several metazoan species are shown in base pair length (bp). The genome version and scaffold number are given for each species, or accession numbers where sequences were obtained from a clone. Table S2: Amphioxus SYCP1 predicted promoter sequences. Sequences for the predicted promoter sequences seen in figure 4 are given, with bold italics denoting the position of predicted transcriptional start site (TSS) where possible. Table S3: Metazoan SYCP1 sequences. Sequences collected for protein alignment and phylogeny are listed. The species is given with the common name in brackets, along with the phylogenetic grouping of the species. Finally accession numbers for all sequences used are given. (PDF 16315 kb) [file 427_2017_600_MOESM1_ESM.pdf]

Supplementary Material

Title: Amphioxus *SYCP1*: a case of retrogene replacement and co-option of regulatory elements adjacent to the ParaHox cluster

Journal: Development, Genes and Evolution

Authors: Myles G. Garstang* and David E.K. Ferrier

Affiliation: The Scottish Oceans Institute, Gatty Marine Laboratory, University of St Andrews, East Sands, St Andrews, Fife, KY16 8LB, UK.

*** Current address: School of Biological Sciences, University of Essex, Wivenhoe, Colchester, Essex, CO4 3SQ**

Corresponding author: David E.K. Ferrier
The Scottish Oceans Institute, Gatty Marine Laboratory, University of St Andrews, East Sands, St Andrews, Fife, KY16 8LB, UK.
Tel.: +44 (0)1334 463480
e-mail: dekf@st-andrews.ac.uk

Figure S1: Full-length protein alignment of metazoan SYCP1 proteins.

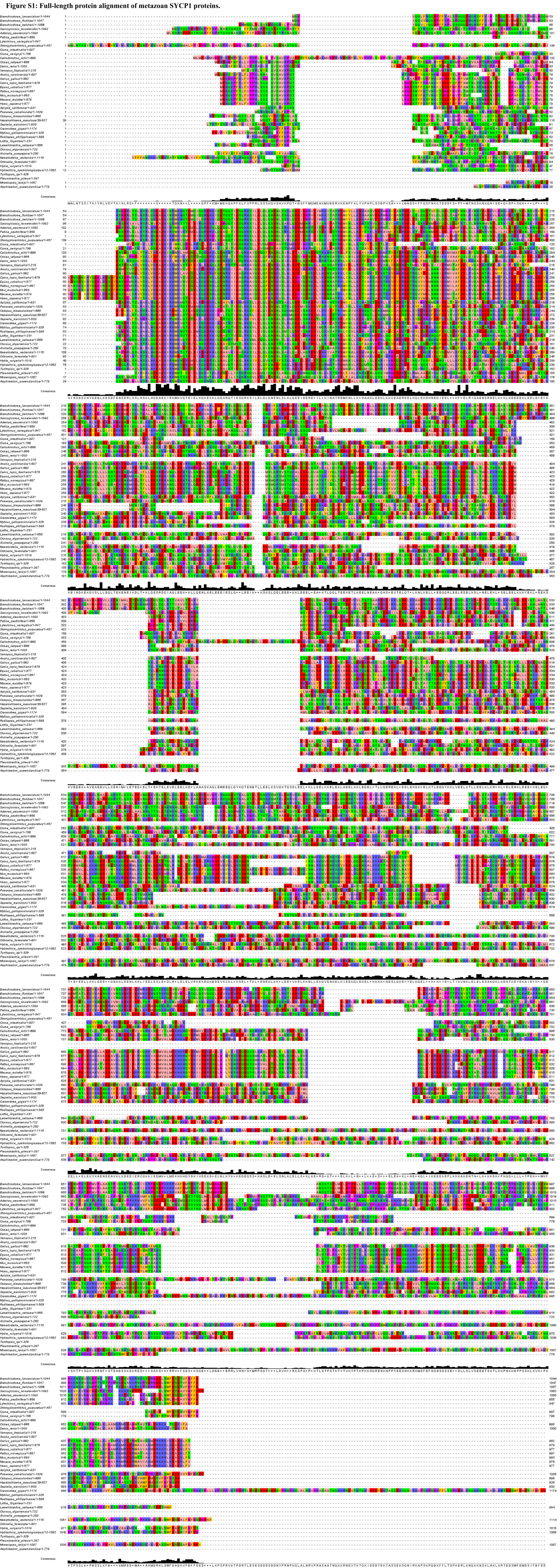

Table S1. Metazoan SYCP1 intron length comparison

Species (Genome version)	No. of *SCP1* introns	Length of transcribed *SCP1* gene (bp)	Total intron + exon length (bp)	Scaffold/Chromosome
Mus musculus (GRCm38)	33	3437	117601	Chr3: 102,818,499-102,936,100
Homo sapiens (GRCh38)	32	3452	140567	Chr1: 114,854,803-114,995,370
Danio rerio (GRCz10)	32	3440	8345	Chr6: 49,054,088-49,062,433
Callorhinchus milii (ESHARK1)	24	2462	30416	scaffold_89: 538,459-573,196
Ciona intestinalis (Cioin2)	0	2696	2696	chr_08q:4728476-4808970
Branchiostoma floridae	0	3141	3141	PACS 33B4, 36D2
Strongylocentrotus purpuratus (Spur_v3.1)	23	2816	11135	Scaffold2806:1-29859
Patiria Miniata (Pmin_v1)	22	1668	12900	Scaffold2247:1-47700
Aplysia californica (Broad 2.0/aplCal1)	21	2196	18402	Scaffold_829:1-266,202
Capitella teleta (Capca1)	25	3048	5745	scaffold_46:228355-395154
Lottia gigantea (Lotigi1)	21	2214	15448	sca_59:503750-573256
Hydra vulgaris (Hydra_RP_1.0)	6	3056	26989	HYDRAscaffold_36716
Amphimedon queenslandica (Aqu1)	23	2331	4812	Scaffold Contig13414: 96,588-101,400

Table S2. Amphioxus *SYCP1* predicted promoter sequences

B.floridae	
**Identifier**	**Sequence (Bold italic font indicates predicted TSS)**
NNPP 1	TAGTACAGTGTATAAGAGTCTATTATAACGAAAAAAAATC***T*** GACTTTCTG
NNPP 2	TACAAGTAGGAAAAAAAGCTGCAGAGAAGTCAGAAATCAG***A*** AAGTCAGAT
NNPP 3	TCCCTTTTTCCAAAAATGGCGCCTCCAAGTCAGAATGTCG***T*** CTGCAAGTG
NNPP 4	TATGTGTAGATATATGAATTGCGATCACGCCTAGCGTAGC***A*** AAGTATTGT
NNPP 5	ATCTATTGTTCATAAAAGGAAACGAACCCCCTTGACACTC***A*** CCAAAGGCA
TSSW Promoter TSS	TCACTTGCAGACGACATTCTGACTT***G*** GAGGCGCCATTTTTGGAAAAAGGG
ProScan 1	CCATAAGCCGCTCATCTTCGTCATCTTCCTCGTAAATCGCATCGAAGTCCGCCATGATGGAAACATCTGCG<br>CACGGTATGCTGGGAAATATCTCCCGGGGTGCAGTTCGAGGTGACGTCACTTGCAGACGACATTCTGACT<br>TGGAGGCGCCATTTTTGGAAAAAGGGAGTTTCTTCCAACAAGAACTGTAAGTGACCTCCTGTTAATCCAGT<br>TCTCTATGTGTATGTAGTTCTGTTAATATCGTAATACA
Proscan 2	ATATCTCCCGGGGTGCAGTTCGAGGTGACGTCACTTGCAGACGACATTCTGACTTGGAGGCGCCATTTTT<br>GGAAAAAGGGAGTTTCTTCCAACAAGAACTGTAAGTGACCTCCTGTTAATCCAGTTCTCTATGTGTATGTA<br>GTTCTGTTAATATCGTAATACAAACATCTTAACGCGTTAAAATTATTTTTTCGGTGGGAGGGCGAA
B.lanceolatum	
**Identifier**	**Sequence (Bold italic font indicates predicted TSS)**
NNPP1	ATTCTACATGTATTAAAAATACCTACTACACGATAGGGAC***A*** CTTTGCAGA
NNPP2	CCCTACATTTTATATAGACATGCCTGCTTGAATTGTTTGG***T*** TCAGACGCA
NNPP3	CAGGCATGTCTATATAAAATGTAGGGTGAAAAGGTCTTTT***T*** TTATGTCCT
NNPP4	GCATGGAGGACATAAAAAAAGACCTTTTCACCCTACATTT***T*** ATATAGACA
NNPP5	CTCCTCTCTGCAAAAATGGCGCCTCCGAGTCAAAATGTCG***T*** CTGCAAGTG
NNPP6	CACTGAATCTAAATATAACAAGTTACCGCAACTCGTATTC***A*** GACACATAT
NNPP7	TCTTTTGTTCTTAAAAAGGACATGAACTCCTGCTGACACT***C*** CTTATAAGG
NNPP8	CTGACACTCCTTATAAGGCAGCGGATTTGAGGTGTGCGGT***A*** TAGATAAAG
NNPP9	ACACTTACTTTATCTATACCGCACACCTCAAATCCGCTGC***C*** TTATAAGGA
TSSW Promoter 1 TSS	GGCATACCGTGCGCAG***A*** TGTTTCCATCATG
TSSW Promoter 2 TSS	GAGTCAAAATGTCGTC***T*** GCAAGTGACGTCA
Proscan1	ACGCGTTGATATGTTTTTATTCCGATGCTAGTCTGATACATTAAGTCATACATAGCGAACTGGAATAACAG<br>TAGATCACTTACTTCTTGGGAGAAAAATCCCTGAAGAAAAATCCCTCTGTTCCTCCTCTCTGCAAAAATGG<br>CGCCTCCGAGTCAAAATGTCGTCTGCAAGTGACGTCACTTCGAACTGCACCCCGGGAGATATTTCCCGGC<br>ATACCGTGCGCAGATGTTTCCATCATGGCGGACTTCGA

Table S3. Metazoan SYCP1 sequences

Species	Group	Accession number/ ID
Alvinella pompejana (Pompeii worm)	Lophotrochozoa-Polychaeta	GO222799.1
Amphimedon queenslandica (demosponge)	Porifera	ACUQ01003074.1
Anolis carolinensis (lizard)	Tetrapoda	XP_008108235.1
Aplysia californica (Sea hare)	Lophotrochozoa-Gastropoda	XP_005108708.1
Asterias amurensis (northern pacific sea star)	Echinodermata-Asteroidia	GAVL01043045.1
Branchiostoma floridae (amphioxus)	Chordata-Cephalochordata	AC129948, AC129947
Branchiostoma lanceolatum (amphioxus)	Chordata-Cephalochordata	Bl71nemh Sc0000037:73,417-80,768, Unpublished cDNA
Branchiostoma belcheri (amphioxus)	Chordata-Cephalochordata	XM_019769777.1
Callorhinchus milii (elephant shark)	Chordata-Chimaera	XP_007899851.1
Canis lupis familiaris (dog)	Chordata-Tetrapoda	XP_857086.1
Ciona intestinalis (Sea Squirt)	Chordata-Tunicata	KH2012:KH.C8.516.v1.A.ND1-1
Ciona savignyi (Sea Squirt)	Chordata-Tunicata	AACT01000684.1
Crassostrea gigas (pacific oyster)	Lophotrochozoa--Bivalvia	XP_011438578.1
Danio rerio (zebrafish)	Chordata-Actinopterigii	NP_001112366.1
Equus caballus (horse)	Chordata-Tetrapoda	XP_001496166.2
Gallus gallus (chicken)	Chordata-Tetrapoda	XP_004935063.1
Hapalochlaena maculosa (southern blue-ringed octopus)	Lophotrochozoa-Cephalopoda	GEXH01115743.1
Homo sapiens (human)	Chordata-Tetrapoda	EAW56621.1
Hydra vulgaris (freshwater hydroid)	Cnidaria-Hydrozoa	JQ906934.1
Hydractinia symbiolongicarpus (marine hydroid)	Cnidaria-Hydrozoa	GCHW01005973.1|
Lamellibrachia satsuma (Giant tube worm)	Lophotrochozoa-Polychaeta	GEHO01210685.1
Lottia Gigantea (owl limpet)	Lophotrochozoa-Bivalva	FC693207.1
Lytechinus variegatus (green sea urchin)	Echinodermata-Echinoidea	GAUR01060760.1
Macaca mulatta (rhesus macaque)	Chordata-Tetrapoda	XP_001111808.1
Mnemiopsis leidyi (sea Walnut)	Ctenophora	GFAT01124492
Mus musculus (mouse)	Chordata-Tetrapoda	CAA86262.1
Mytilus galloprovincialis (mediterranean mussel)	Lophotrochozoa--Bivalvia	GAEN01007290.1
Nematostella vectensis (starlet sea anemone)	Cnidaria-Anthozoa	HADP01191031.1
**Species**	**Group**	**Accession number/Scaffold ID**

Octopus bimaculoides (california two-spot octopus)	Lophotrochozoa-Cephalopoda	XM_014935107.1
Olavius algarvensis (gutless oligochaete worm)	Lophotrochozoa-Oligochaeta	HACZ01084717.1
Orbicella faveolata (mountainous star coral)	Cnidaria-Anthozoa	XM_020759056.1
Orzias latipes (medaka)	Chordata-Actinopterigii	JQ906936.1
Patiria pectinifera (blue bat star)	Echinodermata-Asteroidia	GAVT01095647.1
Pleurobrachia pileus (sea gooseberry)	Ctenophora	FP999277
Pomacea canaliculate (channelled applesnail)	Lophotrochozoa-Gastropoda	GBZZ01052649.1
Rattus norvegicus (rat)	Chordata-Tetrapoda	NM_012810.1
Ruditapes philippinarum (littleneck clam)	Lophotrochozoa--Bivalvia	JO101748.1
Saccoglossus kowalevskii (acorn worm)	hemichordata	XM_006821456.1
Sepiella maindroni (cuttlefish)	Lophotrochozoa-Cephalopoda	GFLT01025932.1
Strongylocentrotus purpuratus (purple sea urchin)	Echinodermata-Echinoidea	XM_011679322.1
Turritopsis sp. (immortal jellyfish)	Cnidaria-Hydrozoa	IAAF01017580.1
Xenopus tropicalis (western clawed frog)	Chordata-Tetrapoda	XP_012811338.1
CCDC39-Drosophila Melanogaster	Ecdysozoa-Hexapoda	ACD81657.1
CCDC39-Homo Sapiens	Chordata-Tetrapoda	NP_852091.1
CCDC39-Strongylocentrotus purpuratus	Echinodermata-Echinoidea	XP_781717.3
